# Gastric Intestinal Metaplasia and Its Rapid Progression Toward Gastric Adenocarcinoma: A Call for Clear Patient Management and Awareness

**DOI:** 10.7759/cureus.18751

**Published:** 2021-10-13

**Authors:** Hussam Al Hennawi, Anwar Khedr, Ramanpreet K Maan, Mohieddin Albarazi, Purna Atluri

**Affiliations:** 1 Internal Medicine, Alfaisal University College of Medicine, Riyadh, SAU; 2 Internal Medicine, Tanta University Faculty of Medicine, Tanta, EGY; 3 Medicine, Lady Hardinge Medical College, New Delhi, IND; 4 Gastroenterology, University Hospital of Brooklyn, State University of New York Downstate Medical Center, Brooklyn, USA

**Keywords:** esophagogastroduodenoscopy (egd), h pylori infection, gastric adenocarcinoma, gastric malignancy, gastric intestinal metaplasia

## Abstract

Gastric cancer is one of the leading causes of cancer-related death worldwide. *Helicobacter pylori* (*H. pylori*) infection is known to cause gastric adenocarcinoma in a stepwise fashion. Gastric intestinal metaplasia is a known premalignant stage. We report a case of a 70-year-old male patient with active chronic *H. pylori-*associated gastritis and focal intestinal metaplasia on the initial presentation, who rapidly developed diffuse, poorly differentiated gastric adenocarcinoma 20 months after the loss to follow-up. Our case highlights the premalignant nature of gastric intestinal metaplasia (GIM) and the extreme importance of early eradication of *H. pylori*. We also address the lack of definitive GIM surveillance guidelines.

## Introduction

Gastric cancer constitutes the fourth most common malignancy associated with high mortality risk and ranks the fifth most common cancer in terms of worldwide incidents [1]. The causation of gastric cancer is multi-factorial, involving the interplay between host genetic factors, bacterial virulence factors, and environmental triggers; nevertheless, knowledge is still lacking on the exact mechanisms underlying the development and progression of gastric cancer [2]. Owing to the aggressive nature of such malignancy, no effective modalities of treatment have been identified to cure gastric cancer. For this reason, prevention remains the most effective strategy to decrease gastric cancer incidence worldwide and abate mortality rate.

The Lauren classification divides gastric adenocarcinoma according to histopathological findings into intestinal and diffuse types [3]. The diffuse-type gastric cancer is more aggressive and tends to present at an advanced stage stemming from molecular defects in cellular adhesions. On the other hand, intestinal-type gastric cancer involves a cascade of interactions between host genetics, environment, and pathogen [4]. Nevertheless, *Helicobacter pylori* (*H. pylori*) infection triggers an inflammatory process, which is the strongest risk factor for both types of gastric cancers. Resulting from the chronic inflammatory process by *H. pylori*, gastric intestinal metaplasia (GIM) constitutes a precancerous lesion resulting in intestinal-type gastric cancer [5].

In this case report, we highlight the consequence of delayed *H. pylori* eradication treatment in patients with GIM and how this might lead to an accelerated progression toward the development of gastric adenocarcinoma. We also bring to light the lack of multi-societal definitive guidelines established for determining the intervals for endoscopic surveillance in patients with GIM.

## Case presentation

**Figure 1:**
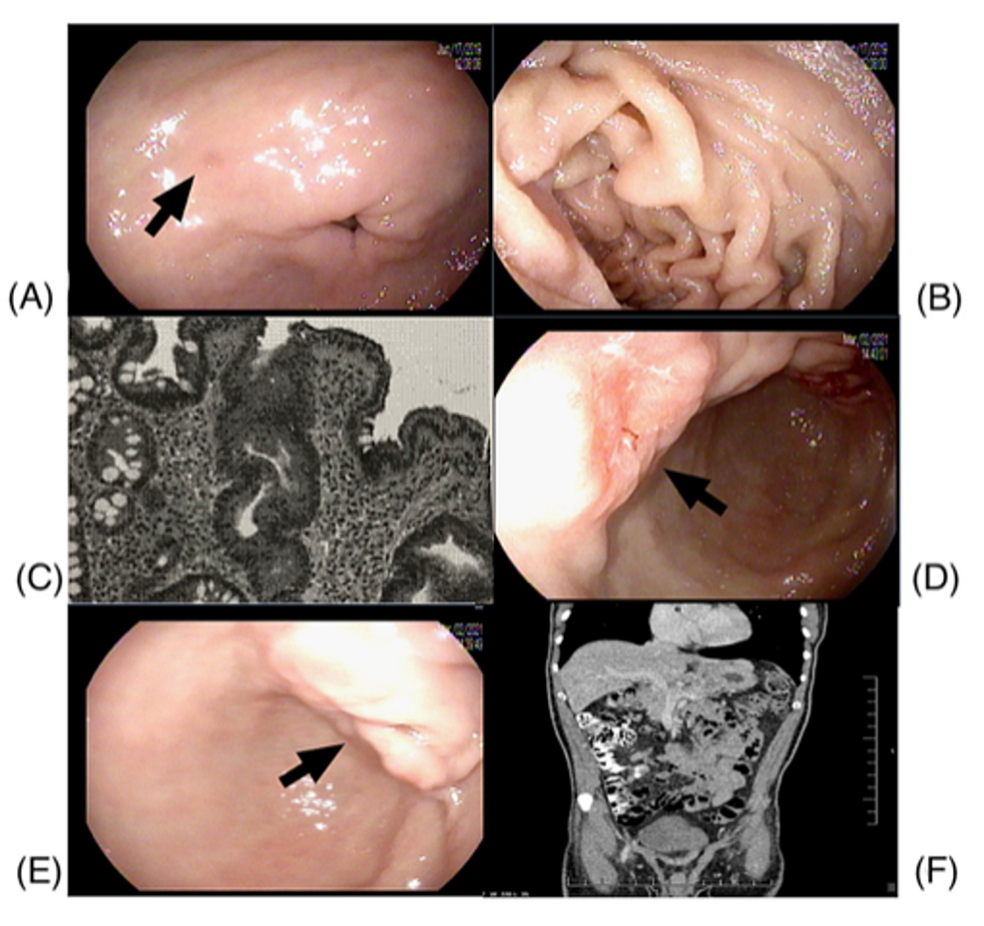
EGD, pathology, and imaging workup EGD showing fragments of localized erythematous antral spots (A, B). Pathology workup showing focal antral intestinal metaplasia on Alcian Blue/PAS stain (C). EGD showing erythematous edematous mucosa along the lesser curvature and the circular fold (D, E). CT of the abdomen and pelvis with IV contrast showing diffuse gastric wall and peritoneal soft tissue thickening adjacent to the lesser curvature with no signs of metastasis (F). EGD: esophagogastroduodenoscopy, CT: computerized tomography, IV: intravenous.

A 70-year-old African American male with a medical history of hypertension, type 2 diabetes mellitus, hyperlipidemia, and obesity presented to the outpatient clinic in June 2019 with upper abdominal discomfort, recurrent vomiting, and loss of appetite. The patient was evaluated for dyspepsia accordingly. Esophagogastroduodenoscopy (EGD) was performed and showed fragments of localized erythematous antral spots (Figure 1A, 1B). The patient was prescribed omeprazole 40 mg. The biopsy returned positive for *H. pylori* gastritis and focal intestinal metaplasia (Figure 1C) at the antrum with no identified dysplastic changes on the Alcian Blue/PAS stain (AmeriPath Northeast, Connecticut, USA). Despite repeated efforts to contact the patient, he did not return for follow-up. 

Twenty months later, the patient returned with worsening upper abdominal discomfort, persistent vomiting, and loss of appetite. He denied melena, bright red bleeding per rectum, and weight loss. His physical exam was unremarkable. The patient underwent repeat EGD and multiple biopsies. EGD showed erythematous edematous mucosa along the lesser curvature and the circular fold (Figure 1D, 1E). Given the patient’s previous biopsy results and prior medication noncompliance, an eradication treatment consisting of amoxicillin/clarithromycin and a proton pump inhibitor was prescribed accordingly after the procedure. Antral biopsy showed active chronic *H. pylori* gastritis and poorly differentiated adenomatous cancer on a background of intestinal metaplasia. Two distinct gastric body biopsies showed chronic active *H. pylori* gastritis and poorly differentiated gastric adenomatous cancer with no intestinal metaplasia on Alcian Blue stain. All biopsies turned positive for anti-cytokeratin monoclonal antibodies (AE1/AE2) on immunohistochemical studies. Computerized tomography (CT) of the abdomen and pelvis with intravenous (IV) contrast showed diffuse gastric wall and peritoneal soft tissue thickening adjacent to the lesser curvature with no signs of metastasis (Figure 1F). CT chest with IV contrast showed no evidence of intrathoracic metastatic disease. The patient was then referred to oncology for further management.

## Discussion

GIM is a precancerous lesion that represents an increased risk of the development of intestinal-type gastric adenocarcinoma. The most common cause is *H. pylori* infection. In GIM, the mucosal lining of the stomach changes into an intestinal-type columnar epithelium [6,7]. There is a wide variation in the reporting of GIM prevalence in large databases, which varies from 3.4% to 29.6%. GIM incidence also varies by country, as shown in some Western and Asian countries in Table 1 [8-10]. GIM can be found on routine biopsies of normal mucosa or targeted biopsies of abnormal mucosal lesions [11].

**Table 1:** Reported incidence of gastric intestinal metaplasia worldwide

Country	Incidence
USA	7.4%–19%
Colombia	25.7%
Germany	22.9%
Netherlands	25.3%
Italy	12.7%–32.4%
China	29.3%
Japan	37%

The Correa cascade describes the series of events where normal gastric mucosa turns into intestinal-type adenocarcinoma. The process begins with chronic inflammation due to *H. pylori* or other causes, and then non-atrophic gastritis, which may persist or advance into atrophic gastritis. Atrophic gastritis is considered to be the first step in the precancerous cascade, which is followed by GIM; then this progresses to low-grade and then high-grade dysplasia and finally forms invasive carcinoma [12].

The link between *H. pylori* and GIM has been long established. One of the critical questions in our case is whether treating *H. pylori* would prevent or slow the progression of GIM or if GIM is a no-return point. There are some studies that support the former theory. In a single-blinded uncontrolled prospective trial by Ohkusa et al., antral intestinal metaplasia improved in 61% of the patients who already had GIM and also improved the inflammation and glandular atrophy [13]. Another study by Sung et al. demonstrated that *H. pylori* eradication led to a significant decrease in gastric inflammation and antral GIM activity [14]. However, some systematic reviews showed no significant difference in GIM before and after *H. pylori* eradication. A meta-analysis by Wang et al. showed no significant difference in antral GIM before and after *H. pylori* eradication [2]. Generally, the consensus is that *H. pylori* should be tested for and treated in patients with GIM due to the established link between *H. pylori* and gastric cancer [2]. Unfortunately, our patient did not receive any treatment for *H. pylori*.

Until two years ago, there were no guidelines regarding the screening of gastric cancer in patients with GIM in the United States. Management differed on a case-by-case basis and by the physician’s opinion [6]. However, the European Society of Gastrointestinal Endoscopy puts some recommendations regarding the surveillance of GIM. It recommends surveillance every three years for patients with focal intestinal metaplasia after eradicating *H. pylori* [15]. But in patients like those who did not complete *H. pylori* eradication or were resistant to the treatment, there are no guidelines about the time intervals for screening despite the possibility of increased incidence. The cost-effectiveness remains a significant issue when considering surveillance for GIM. In a study conducted by Yeh et al., they found that surveillance for GIM every 10 years led to decreasing lifetime cancer risk by 61%. However, this surveillance costs $544,500 per quality-adjusted life-year [16]. They also found that endoscopic surveillance for lesions less advanced than dysplasia was not cost-effective except in high-risk ethnicities [16].

In 2019, the American Gastroenterological Association (AGA) released its recommendations regarding the management of GIM [17]. They also synthesized a clinical decision support tool [18]. They recommended testing for *H. pylori*, followed by eradication if present and confirmation of eradication, in patients with GIM. However, the AGA recommended against the routine use of endoscopic surveillance and short-term repeat endoscopy for risk stratification, except in certain cases with a high risk of gastric cancer. The high-risk conditions include a family history of gastric cancer, especially in a first-degree relative, racial or ethnic minorities, immigrants from countries with a high incidence of gastric cancer, and an incomplete extensive histological type of GIM. The AGA encouraged shared decision-making between clinicians and patients regarding the use of surveillance endoscopy and balancing the benefits and risks for each patient [17].

## Conclusions

Thus far, there is no consensus regarding the endoscopic surveillance interval of *H. pylori*-associated GIM. Some studies have shown evidence in support of surveillance upper endoscopy for GIM patients, which may lead to early detection of gastric adenocarcinoma and improved survival. However, different guidelines have suggested surveillance endoscopy at different time intervals. Although eradication therapy does not reverse GIM, it may hinder the rapid progression to gastric adenocarcinoma. Our patient did not receive proper *H. pylori* eradication treatment and surveillance endoscopy, which could have potentially reduced his risk of progression to gastric adenocarcinoma.

Although complicated by poor patient compliance, our case highlights the malignant nature of GIM and the need for more education to increase patients’ cancer awareness. Additionally, more studies are warranted to recommend the optimal time interval for surveillance endoscopy in patients with GIM.
